# Stroke in Older Survivors of Ischemic Stroke: Standard Care or Something Different?

**DOI:** 10.3390/geriatrics2020018

**Published:** 2017-06-19

**Authors:** Isobel J. Hubbard, Suzanne Wass, Elizabeth Pepper

**Affiliations:** 1 School of Medicine and Public Health, University of Newcastle, Newcastle, NSW 2305, Australia; 2 Neurology Department, Calvary Mater Hospital, Newcastle, NSW 2293, Australia; Suzanne.Wass@calvarymater.org.au (S.W.); Elizabeth.Pepper@calvarymater.org.au (E.P.)

**Keywords:** stroke, ageing, secondary prevention and rehabilitation

## Abstract

Stroke is one of the leading causes of death and disability and it is more likely to occur in those who are older. Because people are living longer, the definition of “old” continues to evolve. Age alone should not influence the healthcare that a patient receives, however, evidence indicates that this does occur, especially in older patients. On the basis of the available evidence, it is time to reconsider whether or not stroke care should differ in older survivors of stroke and if so, why. This is a narrative review of stroke-related health care in those with a recent ischemic stroke. It seeks to answer the following question: Should patients aged ≥80 years who have experienced a recent ischemic stroke receive standard care or something different, and if they should receive something different, what should they receive and why? The review focusses on long-term survival, hyper-acute care, secondary prevention, and rehabilitation. The authors propose a number of recommendations in relation to stroke care in older survivors of a recent ischemic stroke.

## 1. Introduction

Stroke is one of the leading causes of death and disability in adults and its global burden of disease is significant. The risk of stroke increases with age [1] and in nations with aging populations, strokes are occurring in increasingly older people. This has resulted in new definitions of “old” and new paradigms to the age of those receiving stroke care and/or recruited into stroke studies and clinical trials. In the context of equitable health care across populations, a patient’s age should not influence the stroke care that he/she receives. However, when compared to their younger counterparts, there is evidence that this does occur in clinical practice, particularly in older patients [2,3,4]. For example, there are calls to “facilitate equal access to specialized stroke care for the elderly” [5]. In the context of health care needs specific to certain populations, there is evidence that older patients have different health care needs [6,7]. Therefore, in health care, it is important to balance the ethos of not changing post-stroke management simply on the basis of a patient’s age against evidence indicating that the health care needs of older patients can be different to those in younger patients. This balance must be achieved using evidence-based strategies.

Currently, there are occasions when age is, and is not, a consideration in relation to stroke management. On the one hand, age is not a consideration in most nationally-agreed, stroke-specific clinical guidelines [8,9,10], whereas it has been a limitation in the prescribing of post-stroke thrombolysis [11,12]. Currently, the definitions and paradigms for “older” patients are shifting. Whereas studies investigating long-term survival after stroke used to consider 80 years of age as the “high-water” mark [13,14], more recent studies are increasingly reporting findings in patients 90 years and over [7]. Currently, evidence indicates that when compared to those who have not experienced stroke, older patients with stroke are more likely to die [7] or to stay in hospital longer and/or be discharged to a place that is different from their pre-admission residence [5]. Should this cohort of patients receive standard care or are there times when age should be a consideration? 

In critically appraising the relevant evidence, this review seeks to pique discussion about the impact of age on stroke management and in turn, propose answers to the following questions. In patients with ischemic stroke who are ≥80 years of age, are there notable clinical scenarios when age:

1. Should influence their health care and why?
2. Should *not* influence their health care and why?

Although the authors acknowledge the following issues are important to patient care after stroke, this literature review will not systematically search, capture, appraise, and analyze the evidence, nor will it explore the impact of other individual patient characteristics on post-stroke management, nor will it review evidence relating to post-stroke complications. Also, readers should bear in mind that these are review-based recommendations only, and as such, do not replace clinical reasoning and patient-informed decision making.

## 2. Review of the Evidence

### 2.1. Epidemiology: Long-Term Survival and Physical Function

“At a global level, the most rapidly growing age group is the one aged 80 and over” [5]. Where there are ageing populations, there are also trends towards a higher incidence of stroke later in life [1]. In those aged 80 years and over, Russo et al. [15] reported the number of first strokes per 1000 person-years were 17.2 in those who were 80–85 years old, but this increased to 20.8 in those aged 85 years and over. In their analyses of the hospital data of 26,676 Canadian patients with ischemic stroke, Saposnik et al. [5] reported 38% were aged ≥80 years. This sub-cohort of patients was more likely to stay longer in hospital and less likely to be admitted to intensive care and/or go home. During their admission, 24.2% of patients aged >80 years died, compared to only 5.7% in patients aged <60 years. When compared to men and irrespective of age, the investigators found that women were less likely to be admitted to intensive care and less likely to be discharged to their pre-admission place of residence.

In an analysis of longitudinal data in older Australian women, investigators found that, when compared to those who did not report stroke, the long-term survival in women who reported stroke was significantly reduced, even after adjusting for comorbidities [7]. Additional lifestyle risk factors for mortality included obesity and past smoking. However, in a second study investigating the same dataset [16], findings revealed that the “10-year mortality rate was 37% for women with stroke and adequate physical function, and 51% for women with stroke and poor physical functioning”. This indicates that many older women are living with stroke for more than 10 years and many are doing so with poor physical function. 

**Recommendation:** Although stroke is associated with an increased risk of mortality, health care professionals and providers should plan for, and assume, that patients with stroke who are aged ≥80 years could live for another 10 years.

### 2.2. Hyper-Acute Management

In the first few hours following symptoms, organized Stroke Unit care continues to be an effective intervention [17] irrespective of age, sub-type, or severity of stroke. Patients who receive organized inpatient care are more likely to survive, regain independence, and return to living at home. In the first few hours post-ischemic-stroke, there are two additional interventions available to patients that have been shown to improve outcomes. The first is post-stroke thrombolysis and the second is endovascular thrombectomy. 

#### 2.2.1. Post-Stroke Thrombolysis

In the first 4.5 h post-event, alteplase, a recombinant tissue-type plasminogen activator (rt-PA), has been shown to significantly improve outcomes [18,19] in patients with ischemic stroke. However, traditional exclusion criteria for thrombolysis are based on clinical trial data which often excludes those over 80 as a matter of course, hence the limited data available for patients in this age group [20]. Advanced age is the second most common reason clinicians withhold thrombolysis [11,12], possibly due to concerns about increased risk of intracranial hemorrhage. This is despite emerging evidence that the efficacy and safety of alteplase are similar in older cohorts. Emberson et al. [18] reported: “Irrespective of age or stroke severity, and despite an increased risk of fatal intracranial hemorrhage during the first few days after treatment, alteplase significantly improves the overall odds of a good stroke outcome when delivered within 4.5 h of stroke onset, with earlier treatment associated with bigger proportional benefits”, a finding supported by Matsuo et al. [20]. A series of smaller studies over the past 3 years [21,22,23,24,25] consistently found no difference in recanalization rate, in-hospital mortality, or incidence of intracranial hemorrhage in patients over 80 years when compared to younger cohorts, although some cautiously used a lower dose of rt-PA in the older group. Poorer outcomes were noted by Busl et al. [26] after administration of rt-PA in elderly patients with pre-existing dementia, possibly due to underlying vascular pathology or amyloid angiopathy. Pre-stroke dementia was found to be an independent predictor of in-patient mortality and poor outcome following ischemic stroke. 

**Recommendation:** In the first 4.5 h post-ischemic stroke, age alone should not influence whether or not a patient receives Stroke Unit care and/or post-stroke thrombolysis. 

#### 2.2.2. Post-Stroke Endovascular Thrombectomy

Until recently, endovascular thrombectomy was only thought to be effective in the first 7 h post-stroke. In 2016, Saver et al.’s meta-analysis [27], reported a benefit to absolute rates of functional independence 3 months post-event in those undergoing intervention. Their meta-analysis captured five studies which recruited about 15% of participants who were ≥80 years of age; however, age was not factored into the analyses. In the year before, Campbell et al. [28] made the following recommendation: “In view of the data available at present, as is the case for intravenous thrombolysis, exclusion of patients from endovascular treatment on the basis of age alone is not justified”. However, in a study not captured in the meta-analysis, Villwock et al. [29] reported the odds of inpatient mortality in those more than 80 years of age to be twice that of younger patients. New evidence from Jovin et al. [30] and the DAWN study group, presented at the recent European Stroke Organisation Conference may pave the way for a dramatic change in the way we manage stroke in the first 24 h. Their international, multicenter, randomized study examined 90 day outcomes in those undergoing mechanical clot retrieval up to 24 h post stroke. Age was not a barrier to inclusion in the trial, with a participant age range of 19–91 years and a mean age 64 ± 16 years. The researchers used neuroimaging to select patients with a small core, but a large area of salvageable brain on magnetic resonance imaging (MRI), who were then randomized to undergo thrombectomy or standard medical management. The results were dramatic, with 48.6% of the intervention arm achieving good functional outcomes at 90 days, compared to only 13.1% of the standard treatment arm, with a calculated number needed to treat of only 2.8. The trial was terminated early due to the positive results, and subgroup analysis for age is not available. It is also worth noting this is an Industry Sponsored Trial.

**Recommendation:** Early results from the Jovin et al. [31] study suggest that endovascular thrombectomy in patients selected via neuroimaging may produce significant benefits in functional outcomes, up to 24 h post event. These benefits appear independent of age, and this should not be a barrier to considering intervention. 

#### 2.2.3. Hemicraniectomy

In 2016, Alexander et al. [31] published a meta-analysis showing the mortality benefit of decompressive hemicraniectomy in large middle cerebral artery stroke. The review included 7 randomized controlled trials (RCTs) comparing conservative (best medical practice) with hemicraniectomy within 96 h of stroke, and examined outcomes of mortality and disability. The RCTs collectively included 338 patients and included one trial specifically for patients over 60 years. Hemicraniectomy significantly increased the likelihood of survival (Relative Risk 2.05, *p* < 0.00001) with equal benefit when adjusted for age. However, surgery also increased the likelihood of being alive with a moderately severe disability (modified Rankin Score of 4 or less, RR 2.25, *p* < 0.0001). Not only would this increase likelihood of social dependence and risk of placement into residential aged care, it may not be aligned with acceptable outcomes for the patient. This was demonstrated in the ORACLE Stroke study [32] which examined attitudes towards decompressive hemicraniectomy and desirable outcomes, where most participants felt survival with severe disability and dependency to be unacceptable.

**Recommendation:** No absolute recommendation for hemicraniectomy can be made for older people based on the evidence available. We do, however, recommend that when making decisions around decompressive surgery, the patients individual circumstances and values are taken into consideration before proceeding. 

### 2.3. Preventing Recurrent Stroke

The risk of recurrent stroke is greatest in the first 6 months following an initial presentation [33] making secondary prevention measures an essential part of stroke care. Despite this, older patients often receive less aggressive secondary prevention strategies than younger patients [34], contrary to the evidence that most secondary prevention strategies can be effective in the elderly.

#### 2.3.1. Lifestyle Changes

Patients with behavioral risk factors such as smoking, obesity, excessive alcohol, and low activity have an increased risk of stroke recurrence [35]. Strategies to modify these behaviors can be successful [36,37], and the assumption that elderly patients are not interested is unjustified [38]. Redfern et al. [39] demonstrated that older patients were just as likely to change behavioral risk factors after stroke as younger patients. Weight loss strategies in the elderly should focus on activity levels and strength building exercise, rather than more traditional strategies on calorific restriction, due to concerns of further reduction in muscle mass, and the risks of sarcopenia, frailty and nutritional deficiency [40].

**Recommendation:** Older people should have equal opportunities to participate in lifestyle and behavioral modification programs post stroke. 

#### 2.3.2. Antiplatelet Therapy

All patients with non-cardioembolic, ischemic stroke should be considered for antiplatelet therapy, because it is shown to reduce risk of major cardiovascular events by 22% [41]. Anticoagulant therapy is not recommended in those with non-cardioembolic ischemic stroke due to the increased risk of bleeding, based on the results of the WARS Study [42,43]. This study compared aspirin to warfarin in patients aged up to 85 years. When compared to younger cohorts, aspirin use in the elderly was not associated with any significant difference in efficacy or risk of adverse events. Alternative antiplatelet therapy with clopidogrel may be considered. The CAPRIE study [44], which investigated outcomes in participants with an average age of 62.5 years, showed equal, if not slightly superior, efficacy to aspirin with no significant increased risk of bleeding. There is no evidence for a combination therapy of aspirin and clopidogrel, which has been shown to increase the risk of hemorrhagic stroke [45]. Once again, the average age of trial participant was young (66 ± 10 years) but the results of each may be extrapolated into aging populations. Combination therapy with dipyridamole and aspirin has been shown to significantly reduce all-cause mortality, fatal and non-fatal stroke in patients >65 years, when compared to aspirin alone [46,47]. In the PRoFESS trial [48], which compared dipyridamole plus aspirin to clopidogrel alone, a subgroup analysis of data from those aged over 75 years found no difference in risk reduction between the groups, however, combination therapy was associated with an increased risk of intracranial hemorrhage. 

**Recommendation:** Current trial data supports the use of antiplatelet therapy in older patients, and has demonstrated a comparable relative risk reduction in this cohort, however the extent of the risk of bleeding remains unclear.

#### 2.3.3. Cholesterol Lowering Therapy

There are no RCTs investigating the impact of statins in secondary stroke prevention for the very elderly. There is, however, a small pool of data focusing on the younger elderly group. Subgroup analysis from the observational trial by O’Brien et al. [49] found older patients were less likely to be prescribed a statin when compared to the younger cohort, but a 9% reduction in stroke (*p* = 0.001) was evident at the 2 year follow-up in all age groups. They found no benefit for using high dose over standard dose statins, which may ease some of the concerns clinicians have about the potential risk of adverse effects in the elderly populations. More cautious findings came from several large RCTs which recruited a limited number of elderly participants [50,51,52,53,54]. These studies found consistently significant reductions in the primary endpoints of fatal and non-fatal stroke, but could not support this evidence in the very elderly. Concerns that this age group may be more susceptible to adverse effects of statins, such as myopathy and cognitive decline, may be a barrier to prescribing in older cohorts.

**Recommendations:** Age should not a barrier to cholesterol lowering therapy post stroke, but consideration should be given to lowering the dose to minimize the risk of adverse effects. 

#### 2.3.4. Hypertension

Clinical trials in hypertension have attempted to focus on the older patient and there is a reasonable amount of good-quality evidence available which is summarized in Table 1 [55,56,57,58,59,60]. It is worth noting the limited number of trail participants ≥80 years of age and that most data for this age cohort comes from subgroup analysis. The HYVET trial [60] demonstrated a 30% reduction in fatal and non-fatal stroke, and a 21% reduction in all-cause mortality when hypertension was controlled to an average blood pressure (BP) of 145/76 mmHg (compared to 159/80 with placebo (*p* = 0.001)). Since findings from this trial were published, it has been accepted that BP lowering in the very elderly has significant benefit in the reduction of stroke, however questions remain as to the ideal target systolic BP level in this age group. Support for aggressive lowering of systolic BP was provided by the VALISH [61] and SPRINT [62] trials, with further reductions in all-cause mortality, fatal and non-fatal stroke if the systolic BP <120 mmHg. However, neither study adequately assessed the risk of some of the more common adverse events associated with the very elderly, such as: orthostatic hypotension, falls, electrolyte imbalance, or poor-end organ perfusion which may result in cognitive impairment. These events can also result in quality of life issues, such as functional decline and frailty, which may be unique to older people. In response to the evidence for BP lowering to prevent stroke in the very elderly, the American Heart Association’s 2011 guidelines and the European Society of Hypertension’s 2013 guidelines recommend a target systolic BP of 140–150 mmHg for patients ≥80 years of age [61]. In stark contrast, the Australian Heart Foundation’s 2016 guidelines [62] recommend aggressive systolic BP lowering to targets <120 mmHg in patients ≥80 years of age based solely on the results from the SPRINT study [60]. 

**Recommendation:** Age alone should not be a barrier to moderate lowering of systolic BP in patients at risk of stroke, but caution is recommended for aggressive lowering due to potential adverse effects in elderly patients with multiple co-morbidities, frailty, and polypharmacy.

#### 2.3.5. Anticoagulants in Non-Valvular Atrial Fibrillation and Cardioembolic Stroke

Age is an independent predictor for stroke due to atrial fibrillation (AF), with a yearly risk of >4% and 36% of stroke in those over 80 being due to AF [63]. It has been long established that oral anticoagulation is superior to antiplatelet therapy for secondary prevention in those with non-valvular atrial fibrillation [64]. In 2007 the BAFTA study [65] demonstrated a 52% reduction in fatal or disabling stroke in patients over 75 years with AF treated with warfarin. However, many clinicians remain concerned about potential additional bleeding risks in older populations, complicated prescribing regimes with warfarin, and the need for regular INR checks, which may lead to under-prescribing. The introduction of the direct oral anticoagulants (DOACs) has raised interest in this area, although there are no specific randomized controlled trials comparing warfarin with DOACs in elderly populations, subgroup data from trials is available. In 2014 Sardar et al. [66] published a meta-analysis of subgroup data from 10 RCTs comparing DOACs with warfarin, that collectively included over 25000 elderly patients. They found risk of stroke to be significantly lower with DOACs than with conventional warfarin therapy (3.3% vs. 4.7% OR 0.65 CI = 0.48–0.87; absolute risk reduction 1.4% and number needed to treat of 71). In addition, there was no difference in significant bleeding risk between the groups, however the authors acknowledge that previous articles citing an increase in bleeding risk with DOACs state this is likely due to comorbidities, in particular renal impairment, which may not be reflected in trial data. However, renal dysfunction is not a contraindication for anticoagulation, more an indication that dose adjustment is required and that the clinician should consider yearly monitoring of renal function [67]. Patients with a high risk of falls or cognitive impairment can also be considered for anticoagulation with DOACs, as the benefit of stroke prevention is still shown to outweigh the risks. DOACs have less risk of intracranial hemorrhage, including traumatic intracranial bleeding, in high falls risk populations, when compared with warfarin [68]. The presence of microbleeds on MRI may be a predictor of future intracranial hemorrhage for those considering warfarin. The systematic review by Lovelock et al. [69] included 12 studies and a pooled data set of over 5000 participants. They found microbleeds to be significantly more likely in warfarin uses with an intracerebral hemorrhage compared with non-antithrombotic users (OR 2.7 *p* ≤ 0.001) and when compared to warfarin users with ischemic stroke (OR 8.0, *p* = 0.001). This led them to conclude that the presence of microbleeds on MRI may be a predictor for those patients at risk of future intracerebral hemorrhage and aid decision making when considering warfarin. No similar analysis is available for the DOACs as yet. The decision to prescribe in at risk elderly populations should also consider other factors such as: quality of life, functional status, and additional falls risk such as poorly controlled hypertension, orthostatic hypotension, and visual impairment. DOACs may also be advantageous in older, frailer populations due to the simpler prescribing regime, no requirement for ongoing monitoring, once daily dosage, and less interaction with other drugs. One large scale cohort study [70] and a second observational study [71] support the theory that DOACs are tolerated better in elderly populations, and are less likely to be discontinued (*p* < 0.0001 for all comparisons versus other anticoagulants), particularly in those on once daily dosing regimes.

**Recommendation:** DOACs provide effective prevention from cardioembolic stroke in elderly populations. The presence of co-morbidities, in particular moderate renal impairment, may increase bleeding risk and dose reduction may be required. Age should not be a barrier to anticoagulation in patients with AF.

### 2.4. Sub-Acute Phase: Rehabiliation

In 1994, Alexander [72] found that all patients under the age of 55 years were discharged home irrespective of the severity of the stroke, whereas in those over 55 years, both age and severity influenced discharge destination. However, in that same year, investigators of the Copenhagen Stroke Study recommended that age alone should not influence whether a patient was referred for rehabilitation [73]. In 2008, Denti et al. [74] investigated stroke in patients aged ≥75 years, and found around 80% were discharged home and that rehabilitation in elderly stroke patients could improve function and favorably influence discharge destination. In 2016, similar findings were reported in an investigation of patients aged ≥85 years with 54% being discharged back into the community [75]. Over the past 20–25 years much has changed. People are living longer, and, as a result, the prevalence of first-ever-stroke is increasing. In addition, sub-acute care, including rehabilitation, has become more effective with the introduction of evidence-based practice, clinical guidelines, and national audits [76]. Although age may still influence outcomes, it cannot be assumed that just because a patient is elderly, they are not going to survive for many years [16] and/or benefit from time in stroke recovery programs. Despite this, evidence indicates that, when compared to their younger counterparts, older patients experience higher levels of unmet need in relation to sub-acute stroke and rehabilitation [77]. 

There is evidence of differing needs in older patients with stroke and evidence of differences in outcomes relating to gender [78]. An investigation of the Framingham Study cohort found that, even after adjusting for age and stroke severity, older age accounted for higher levels of disability and that women were more at risk of disability and institutional care. Such evidence demonstrates that in older patients recovering from stroke, consideration needs to be given to their age, gender, and level of disability in their recovery programs. For example, age may influence the intensity of repetitive task-specific training and the meaningful goals in relation to everyday tasks. As with all patients, an individualized approach to recovery after stroke needs to be applied. 

Even in patients with recurrent stroke, Mizrahia et al. [79] reported “elderly patients with recurrent ischemic stroke admitted to a rehabilitation ward, showed similar [Functional Independence Measure] gain scores at discharge, compared with first-ever stroke patients” concluding “that recurrent stroke should not be considered as adversely affecting the short-term functional outcomes of patients in a post-acute rehabilitation setting”.

**Recommendation:** In the first few weeks post-ischemic stroke, age alone should not influence whether or not a patient receives rehabilitation, however, it should be acknowledged that the needs of older people may be different to those of their younger counterparts. 

## 3. Clinical Implications: Standard Care or Something Different?

Ageism exists in stroke care, both in clinical research and “real world” management, which, given stroke is a disease of the elderly, makes evidence based decision making difficult for the clinician. The elderly are excluded from clinical trials for many reasons: presence of co-morbidities which increase the risk of adverse events, cognitive impairment, polypharmacy leading to potential drug interaction, and patient factors such as poor awareness of the importance of trials, mobility and transport issues. Study participants in stroke trials are often 10–15 years younger than those seen in everyday clinical practice [80]. In practical terms, this means many very elderly patients are missing out on standard clinical management due to the perceived lack of evidence. A study from the United Kingdom by Rudd et al. [81] found patients over 85 years were less likely to be admitted to a stroke unit (RR 0.82, 95% CI 0.75–0.90), less likely to receive neuroimaging within 24 h, and less likely to receive secondary prevention or rehabilitation. A cohort study by Raine et al. [82] examined prescribing practices in the United Kingdom for secondary stroke prevention. They found the very elderly (80–89 years) were less likely to receive secondary prevention with an odds ratio of 0.51 when compared with 50–59-year-olds, with the exception of antithrombotic therapy which was more likely to be prescribed (OR 2.92). This was despite a 50% reduction in mortality with appropriate secondary prevention. Encouragingly, in the same year, Saposnik et al. [5] found equality in acute stroke care (including thrombolysis) and mode of delivery across the ages in their Canadian study [82]. They did note, however, that older patients were less likely to have investigation such as carotid imaging, when compared with younger cohorts (*p* = 0.0001). Researchers and clinicians need to embrace good quality stroke care for the elderly. Frailty needs to be quantified and measured during clinical trials, and there should not be age limits for participants. Clinicians need to familiarize themselves with the evidence that is available and consider this, not age alone, when decision making. Where possible, patients and their families should be involved in decision making and clinicians should take into account individual circumstance, function, personal values, and quality of life when caring for the very elderly with stroke. 

Although age is the most important *non-modifiable* risk factor for stroke, age alone should not be used as a reason to withhold or change standard stroke management in those over 80 years. Co-morbidities such as: hypertension, ischemic heart disease, heart failure, and pre-stroke dementia are better predictors of poor outcome, and are independent of age. Clinicians often consider modification of treatment to minimize adverse effects in the elderly but there is limited data to support this practice, especially when it comes to hyper-acute stroke management with thrombolytic agents. Poor management of elderly patients in the early stages of stroke can lead to higher financial and health care burden in the form of increased mortality, morbidity, long term disability, and placement in residential care. A patient-centered approach should be taken to promote the best outcomes for the individual.

## Figures and Tables

**Table 1:** Early trial data and HYVET: evidence for blood pressure (BP) lowering to prevent stroke in the elderly (Systolic BP < 150 mmHg).

Study	Year	Design	Therapeutics	Follow Up	Mean Age	Results
SHEP [55]	1991	RCT Primary prevention	Thiazide diuretic * vs. placebo	5 years	72	36% RR ^ fatal & non-fatal stroke
STOP [56]	1991	RCT Primary prevention	Thiazide diuretic * vs. placebo	2 years	76	40% RR composite endpoint including stroke 43% reduction all-cause mortality
Syst-Eur [57]	1997	RCT Primary prevention	CCB ** vs. placebo	2 years	70	42% RR fatal & non-fatal stroke
INDANA [59]	1999	Subgroup meta-analysis	n/a	n/a	83	34% RR fatal & non-fatal stroke. Increase all-cause mortality ^^
PROGRESS [58]	2001	RCT Secondary prevention	ACE Inhibitor *** vs. placebo	3 years	64	28% RR fatal & non-fatal stroke. Similar benefits for normotensives
HYVET [60]	2008	RCT Primary prevention	Thiazide **** vs. placebo	2 years	84	30% RR fatal & non-fatal stroke 21% reduction all-cause mortality

RCT randomized controlled trial, RR relative risk, ^ risk reduction, ^^ not reaching statistical significance, * plus beta blocker if necessary, ** calcium channel blocker (plus ACE Inhibitor plus beta blocker if necessary), *** plus thiazide if necessary, **** plus ACE Inhibitor if necessary.

## Acronym

**AF**	Atrial fibrillation
**BP**	Blood pressure
**DOACs**	Direct oral anticoagulants
**MRI**	Magnetic resonance imaging
**OR**	Odds ratio
**RCTs**	Randomized controlled trials
**RR**	Relative risk
**rtPA**	Recombinant Tissue plasminogen activator
